# Revision of the genus *Reinmara* Schaus, 1928 (Lepidoptera, Mimallonoidea, Mimallonidae) with the descriptions of four new species from South America

**DOI:** 10.3897/zookeys.677.12435

**Published:** 2017-05-29

**Authors:** Ryan A. St Laurent, Daniel Herbin, Carlos G. C. Mielke

**Affiliations:** 1 McGuire Center for Lepidoptera and Biodiversity, Florida Museum of Natural History, University of Florida, 3215 Hull Road, Gainesville, FL 32611-2710 USA; 2 7, Le Clos de Lutché, F-31380 Garidech, France; 3 Caixa Postal 1206, 84.145-000 Carambeí, Paraná, Brazil

**Keywords:** Bolivia, Brazil, Ecuador, Peru, Taxonomy

## Abstract

The mimallonid genus *Reinmara* Schaus, 1928 is revised. The three previously described species, *R. enthona* (Schaus, 1905), *R. minasa* Schaus, 1928, and *R. wolfei* Herbin & C. Mielke, 2014 are redescribed and the females of each are described and figured for the first time. Additionally, we describe four new species, two Andean: *R. andensis* **sp. n.** and *R. occidentalis* **sp. n.**, and two Brazilian: *R. atlantica* **sp. n.** and *R. ignea* **sp. n.**. The new species *R. ignea* and *R. atlantica* are likely of conservation concern due to their rarity in collections and their apparent endemism to an endangered biome, the Brazilian Atlantic Forest.

## Introduction

The type species of *Reinmara* Schaus, 1928, *R. enthona* (Schaus, 1905), was originally described in *Cicinnus* Blanchard, 1852. *Cicinnus* was, and to some extent, still is a sort of catchall category subsuming many taxa of uncertain phylogenetic position. Later, Schaus (1928) established the groundwork for much of the generic classification in current use for the family. In Schaus’s work, *Reinmara* was described to include the Amazonian *R. enthona* and southeastern Brazilian *R. minasa* Schaus, 1928. Like most mimallonid genera described by Schaus, generic characterization was based primarily on wing venation. However, the close association of *Reinmara* with *Trogoptera* Herrich-Schäffer, [1856], based on male genitalia, was mentioned in this early work.

Since Schaus (1928), one species has been described from the Brazilian Cerrado: *R. wolfei* Herbin & C. Mielke, 2014. Therefore, apart from these two works and the species lists of Mimallonidae (Gaede 1931, Becker 1996), very little about this genus has been published. We here revise this genus, figuring both sexes of the three previously described species, the females of all of which were previously unknown. We also recognize and describe four new species, increasing the known diversity of *Reinmara* to seven species.

## Methods

Dissections were performed as in Lafontaine (2004). Morphological, including genitalia, terminology follows Kristensen (2003). Genitalia and abdomens, when not slide mounted, are preserved in glycerol filled microvials.

Specimens from the following collections were examined:

**AMNH** American Museum of Natural History, New York, New York, USA

**CDH** Coll. Daniel Herbin, Garidech, France

**CEIOC** Entomological Collection of the Oswaldo Cruz Institute, Rio de Janeiro, Rio de Janeiro, Brazil

**CGCM** Coll. Carlos G. C. Mielke, Curitiba, Paraná, Brazil

**CNC** Canadian National Collection of Insects, Arachnids and Nematodes, Ottawa, Ontario, Canada

**CPC** Coll. Philippe Collet, Caen, France

**CUIC** Cornell University Insect Collection, Ithaca, New York, USA

**DZUP** Coll. Pe. Jesus S. Moure, Departamento de Zoologia, Universidade Federal do Paraná, Curitiba, Paraná, Brazil

**ISEZ** The Institute of Systematics and Evolution of Animals of the Polish Academy of Sciences, Kraków, Poland

**MGCL** McGuire Center for Lepidoptera & Biodiversity, Gainesville, Florida, USA

**MNHN** Muséum nationale d’Histoire naturelle de Paris, Paris, France

**MNHU** Museum für Naturkunde der Humboldt-Universität zu Berlin, Germany

**MNRJ** Museu Nacional do Rio do Janeiro, Rio de Janeiro, Rio de Janeiro, Brazil

**MWM** Museum Witt, Munich, Germany

**MZSP** Museu de Zoologia, Universidade de São Paulo, São Paulo, São Paulo, Brazil

**NHMUK** Natural History Museum, London, U.K.

**NHRS** Entomological Collections, Swedish Museum of Natural History, Stockholm, Sweden

**USNM** National Museum of Natural History [formerly United States National Museum], Washington D.C., USA

**VOB** Becker Collection, Camacã, Bahia, Brazil

**ZSM** Zoologische Staatssammlung München [Munich], Germany

Figures were manipulated with Adobe Photoshop CS4 (Adobe 2008). Male genitalia are figured in natural color with CS4 “auto color” used to improve white backgrounds when necessary. The map was built with SimpleMappr (Shorthouse 2010) and edited with CS4. All geographical coordinates are approximate, and are based on the localities provided on specimen labels when coordinates were not explicitly given. GPS data were acquired with Google Earth.

We used DNA barcoding to help distinguish the similar species *Reinmara enthona* from *R. andensis* sp. n. Our barcoding protocol used a standardized short sequence of DNA as a species-level character (Hebert et al. 2003, Stoeckle and Hebert 2008), based on the mitochondrial cytochrome c oxidase 1 gene region (“COI”). These “barcodes” were obtained thanks to IBOL (International Barcoding of Life project), and were used in addition to adult habitus and genitalia examination to differentiate the aforementioned morphologically similar species. The Neighbor-joining method (Saitou and Nei 1987) was used to infer the relationships among sampled *Reinmara* specimens in MEGA6 (Tamura et al. 2013), based on sequences downloaded from and aligned in BOLD (Barcode of Life). 1000 bootstrap replicates were performed and are shown at the nodes of Fig. 7. Evolutionary distances were computed using the Kimura 2-parameter method (Kimura 1980); units of distance reflect the number of base substitutions per site. All codon positions were included. All positions containing gaps and missing data were eliminated. This component of enhanced species delimitation is reflected in the distance tree in Fig. 7.

The symbol ‡ is used in the text to represent unavailable names in the text (Fletcher and Nye 1982).

## Results and discussion

### Reinmara

Taxon classificationPlantaeLepidopteraMimallonidae

Schaus, 1928: 654.

#### Type species.

*Cicinnus enthona* Schaus, 1905; Schaus 1928: 654, by original designation.

#### Diagnosis.

*Reinmara* can be recognized by the usual contrast between medial and submarginal areas due to diffuse, lighter coloration medially, this coloration, combined with the straight forewing postmedial line, notched tornus, and elongated, slightly falcate forewings (males) distinguish this genus from most other Mimallonidae. The morphologically most similar genus, *Trogoptera* Herrich-Schäffer, [1856], has more rectangular forewings and often displays more earthen tones (except in *T. semililacea* (Dognin, 1916) which is similar in color to some *Reinmara* but can be recognized by extremely long saccular extensions in the male genitalia). Genitalia of *Trogoptera* are very similar to those of *Reinmara*, but the fused gnathos is mesally extended by a singular structure, and is not distally separated as is the same structure in *Reinmara*. Schaus (1928) noted this difference in his description of *Reinmara*.

#### Description.

**Male.** *Head*: Pale beige to brown, eyes very large, occupying more than two-thirds area of head; antenna pale brown, tan, bipectinate to tip with distal fifth of pectinations much shorter; labial palpus reduced, not extending beyond frons, three segmented, second segment roughly half length of first, third segment reduced, barely visible; vestigial proboscis present. *Thorax*: Coloration usually as for head but with additional, often pink, shading. *Legs*: Coloration as for thorax, vestiture thick, long; tibial spurs narrow, very sharp, basal half covered in scales. *Forewing dorsum*: Forewing length: 12.0–23.5 mm, wingspan: 33–43 mm. Triangular, outer margin concave to varying degrees mesally; tornus usually strongly notched, apex may appear somewhat falcate in species with prominently concave outer margin. Ground color various shades of brown, sparsely scattered with dark brown, tiny petiolate scales usually present. Ante- and medial areas nearly always with pale pink or almost silvery scales throughout, submarginal area generally appearing darker than medial area. Presence of antemedial line variable, dark postmedial line preapical, well defined. Discal mark always present as pale splotch, with darker central region faint or very prominent. Fringe coloration variable. *Forewing ventrum*: Similar to dorsum but appearing browner overall due to absence of well-defined ante- and medial pink shading, though some pink shading may be present, especially submarginally. Antemedial line absent, postmedial line reduced to traces in all but *R. ignea* sp. n., discal mark more prominent, darker than on forewing dorsum. *Hindwing dorsum*: Shape more rounded, outer margin convex except for when notch present on anterior margin, patterning as for forewing dorsum, but antemedial line absent, discal mark and postmedial line usually weakly defined. *Hindwing ventrum*: Following same pattern as forewing ventrum. Frenulum a single bristle. *Venation*: Typical of Mimallonidae, but Rs3 + Rs4 quite long stalked. *Abdomen*: Coloration usually as for thorax, but browner, with coppery luster in fresh specimens, fading to pale beige in older material. Vestiture thick, long, distal tip of abdomen with elongated, dark-brown tipped scales. *Genitalia*: Vinculum ovoid, circumscribing a complex diaphragm with four setae-filled sacks, from a ventral perspective: upper two sacs much smaller and outwardly everted with long outwardly extended setae, lower two sacks larger (bottom right sack the largest of the four), lower sacks not outwardly everted, setae of lower sacks extended outward from within sacks. Uncus simple, broad, truncated to varying degrees distally, appearing beak-like laterally. Gnathos robust, proximally rectangular or rounded, with broad, dual mesal extensions that are fused together near base but bifurcate as fingerlike tips distally. Valves broad, short, rounded apically, sacculus accentuated as slight fold with both blunt and sharp projections near distal most portion of fold, length of sharp projection usually asymmetrical when comparing sacculus of both valves. Juxta partially fused to ventrum of phallus, basally juxta as widened lip where affixed to vinculum. Base of relatively small phallus narrower than distal portion, distal half of phallus variable in shape. Vesica very small, globular, with singular, long narrower extension. **Female.** *Head*: As for male but slightly broader; antenna dentate with very small pectinations along entire length of flagellum, except in *R. ignea* sp. n. where antenna more similar to that of male, but with smaller pectinations. *Thorax*: As for male. *Legs*: As for male. *Forewing dorsum*: Forewing length: 12–23 mm, wingspan: 27–43 mm. As for male but much broader, margin convex except for just below apex; tornus strongly notched. Coloration and patterning usually as for male, but see *R. ignea* sp. n. *Forewing ventrum*: Similar to dorsum but appearing browner overall due to absence of well-defined ante- and medial pink shading. Antemedial line absent, postmedial line usually reduced to traces, discal mark more prominent, darker than on forewing dorsum. *Hindwing dorsum*: Similar to forewing dorsum, but notch present on anterior margin, patterning as for forewing dorsum, but antemedial line absent, discal mark and postmedial line usually weakly defined. *Hindwing ventrum*: Following same pattern as forewing ventrum. Frenulum as multiple bristles. *Abdomen*: Similar to that of males but more robust overall. *Genitalia*: Stout, usually robust; tergite VIII forms smooth, posteriorly directed tongue-like extension, VIII heavily sclerotized laterally forming curving plate, which extends outward encircling papillae anales. Apophyses anteriores roughly half-length or equal to that of apophyses posteriores. Lamella ante- and postvaginalis converge as a wide, bowl-like structure. Ductus bursae short, narrow. Corpus bursae small in comparison to robust, heavily sclerotized remainder of genitalia, either bag-like or elongated. Papillae anales broad, rounded, covered in long, fine setae.

#### Remarks.

The genus *Reinmara* is broadly distributed in South America. Prior to this study very little was known about the genus and females were unknown.

Unlike most genera of Mimallonidae studied by us in recent years, *Reinmara* have very homogenous male genitalia with only minor differences between externally distinct species (for example *R. enthona* and *R. minasa*), so we relied heavily on external characters, considering habitat specialization and endemism to specific habitats/biomes as seen in other mimallonid genera, as well as in one case COI barcoding, to differentiate species. We also recognize the close similarity in wing shape and male genitalia morphology between *Reinmara* and *Trogoptera*, but maintain them as separate, valid genera pending ongoing phylogenetic work.

#### Key to species of *Reinmara*

**Table:** 

1	Size in both sexes moderate (forewing length: >16 mm), forewing antemedial line very faint, if present at all; ventrally, postmedial line on all wings weakly defined, outwardly curved, usually interrupted by wing veins; forewing apex not falcate or if so, weak, blunt. Male genitalia: phallus cylindrical, weakly curved (for example Figs 23c, 24c, 25c)	**2**
–	Size in both sexes relatively small (12 mm [♂], 12–16 mm [♀]), forewing antemedial line present, not faint; ventrally postmedial line on all wings essentially as well defined and following the same pattern as on dorsum; forewing apex sharply acute, falcate. Male genitalia: phallus thin, strongly curved, and hook-like in shape (Fig. 30c)	***R. ignea* sp. n.**
2	Male: Forewing postmedial line not outwardly lined with black suffusion from tornus to apex. Female: Forewing postmedial line not inwardly lined with light pink-gray suffusion, medial area pink suffused	**3**
–	Male: Forewing postmedial line outwardly lined with black suffusion from tornus to apex. Female: Forewing postmedial line inwardly lined with light pink-gray suffusion, medial area largely displaying the light brown ground color, not suffused with pink	***R. minasa***
3	Forewing with deep notch at tornus, eastern slopes of the Andes mountains	**4**
–	Forewing smooth at tornus, notch absent, west of Andes	***R. occidentalis* sp. n.**
4	Phallus mostly cylindrical in shape, not distinctly broadened distally, found in the Amazon rainforest, moderate elevations of the Andes mountains, or from the Brazilian Atlantic Forest	**5**
–	Phallus distinctly broadened distally, endemic to the Cerrado of Brazil and adjacent regions of Bolivia	***R. wolfei***
5	Forewing postmedial line notched toward costa at intersection with Rs4; forewing narrowed apically, slightly falcate, distributed in the Amazon rainforest and Brazilian Atlantic Forest	**6**
–	Forewing postmedial line not notched toward costa at intersection with Rs4; forewing not noticeably narrowed apically, though if somewhat truncated, submarginal area still broader than any other *Reinmara* species; endemic to eastern slopes of Andes mountains	***R. andensis* sp. n.**
6	Setae-filled diaphragmal sacks of male genitalia well developed, extending into body cavity well beyond vincular ring. Pinkish gray suffusion generally broadly distributed in medial area of forewing. Broadly distributed in the Amazon rainforest	***R. enthona***
–	Diaphragmal sacks of male genitalia half the size of those in *R. enthona*, sacks hardly extending into body beyond vinculum. Gray suffusion of medial area restricted to apical confluence of postmedial line with costa. Endemic to Brazilian Atlantic Forest, so far known only from Espírito Santo	***R. atlantica* sp. n.**

### Reinmara enthona

Taxon classificationPlantaeLepidopteraMimallonidae

(Schaus, 1905)

Figs 1–5 , 6 , 23 , 31 , 36

Cicinnus enthona Schaus, 1905: 325–326
Reinmara enthona ; Schaus 1928, fig. ♂ 88b
Reinmara enthona ; Gaede 1931
Reinmara enthona ; Becker 1996
Reinmara enthona ; Herbin and Mielke 2014, fig. ♂ 42

#### Type material.

**Holotype**, ♂. **FRENCH GUIANA**: St. Jean, Maroni, F. Guiana/ [Holo]Type, No. 8888, U.S.N.M./ USNM-Mimal: 1059/ Collection Wm Schaus/ *Perophora enthona* Type Schaus/ St Laurent diss.: 11-1-16:3/ (USNM, examined). Type locality: French Guiana, St. Jean du Maroni.

#### Additional material examined.

(67 ♂, 3 ♀ total) **SURINAME**: 2 ♂, Moengo, Boven Cottica River: 26.V.1927, Cornell Univ. Lot 760, Sub 79, 80, St Laurent diss.: 10-25-15:2 (CUIC). 1 ♀, Brokopondo, Brownsberg NP, 490 m, 04°56'55"N, 055°10'55"W, 23:55 h, 28.III.2014, A.J. Hielkema [photographer], At HPL/BL (photo examined, Fig. 6, not collected). **FRENCH GUIANA**: 2 ♂, St. Laurent du Maroni, Cr. Naï, PK 13: 27.XII.1991, L. Sénécaux [leg.] (MNHN). 1 ♂, Rd. to Kaw, Camp Patawa: 25.III. –7.IV.2008, S. Kohll leg., DNA sample ID BC-Her 2691 (CDH). 1 ♂, 1♀, St. Jean du Maroni, Rd. Apatou, km 15: 27.VII.2011, Ph. Collet leg., UV (CPC). 1 ♂, St. Jean du Maroni: ex. Coll. Wm Schaus, USNM-Mimal:1784 (USNM). 1 ♀, Rd. to Kaw, km 37.5 + 2, 4°33.691'N, 52°08.391'W, 200 m: 30.VII–8.VIII.2003, ex. Coll. M. Laguerre, DNA sample ID BC-Her 2707, genitalia prep. D. Herbin ref. H. 1103 (CDH). 1 ♂, Rd. to Kaw, km 45: 17.VII.1991, F. Bénéluz leg., BMNH(E) 2008-107, NHMUK010247865 (NHMUK). 1 ♂, Rd. to Kaw: 16–30.XII.1998, A. Le Flao leg. (MNHN). 1 ♂, Rd. to Kaw, km 47: 4.VIII.1991, F. Bénéluz leg., BMNH(E) 2008-107, NHMUK010588025 (NHMUK). 1 ♂, Rd. to Patagai, 5°20'34.23"N, 53°12'47.86"W, 58 m: 3.X.2013, D. Herbin & O. Felis leg. (CDH). 2 ♂, Rd. Patagai/Counamama, 5°20'34.23"N, 53°12'47.86"W, 58 m: 4.XII.2013, D. Herbin leg. (CDH). 1 ♂, Rd. Patagai/Counamama, 5°22'59.51"N, 53°12'27.25"W, 49 m: 23.XI.2013, D. Herbin leg. (CDH). 1 ♂, Saül, Point de vue: 29.VII.2011, Ph. Collet leg., UV (CPC). 1 ♂, Réserve de la Trinité, Aya Haute Koursibo: 7.XI.2013, E. Poirier leg., UV (CPC). 1 ♂, Mont Mitaraka, 300 m: 20.VIII.2015, La Planète Revisitée, MNHN-PNI, Guyane 2015, APA-973-1, Ph. Collet leg. (CPC). 1 ♂, Rd. Changement, km 7: 13.VIII.1991, F. Bénéluz leg., BMNH(E) 2008-107, NMHUK010247866 (NHMUK). 1 ♂, Nouragues, Pararé, 4.038113952°N, 52.67309734°W: 23.V.–5.VI.2014, J. Barber, N. Homziack, A.Y. Kawahara, A. Keener & B. Leavell leg., DNA voucher number LEP-34752 (MGCL, molecular collection, barcoded). 2 ♂, Rd. Apatou, Layons km 26, 5°14'46.31"N, 54°11'07.55"W, 126 m: 1.X.2013, D. Herbin & O. Felis leg. (CDH). 1 ♂, Rd. Apatou, Layons km 26, 5°14'46.38"N, 54°11'52.16"W, 99 m: 2.X.2013, D. Herbin & O. Felis leg. (CDH). 1 ♂, Plateau des Mines, 5°20'42.59"N, 53°4'31.96"W, 49 m: 4.X.2015, D. Herbin & M. Laguerre leg. (CDH). 1 ♂, Rd. Coralie, 4°29'07.43"N, 52°23'49.40"W, 40 m: 7.XII.2013, D. Herbin leg. (CDH). 1 ♂, Rd. Coralie, 4°29'07.43"N, 52°23'49.40"W, 40 m: 7.XII.2013, D. Herbin leg. (CDH). 3 ♂, Rd. Coralie, PK 2: IV.1993, J. Navatte, H. de Toulgoët (MNHN). 1 ♂, Roura, Rd. Coralie, PK 2: 10.XII.1991, P. Kindl, L. Sénécaux leg., Coll. P. Kindl (MNHN). 1 ♂, Surroundings of Coralie, rd. dégrad Correze, PK 0.1: 9.XII.1994, P. Kindl leg., Muséum Paris don de Th. Kindl (MNHN). 2 ♂, Roura, Coralie, Rd. of dégrad Corrèz, PK 0.1, P. 10.XII.1991, 16.IV.1994, Kindl leg., Muséum Paris don de Th. Kindl (MNHN). 2 ♂, Rd. de la Montagne de Fer, 5°20'21.17"N, 53°32'22.10"W, 88 m: 30.IX.2013, D. Herbin & O. Felis leg. (CDH). 2 ♂, Rd. de Kaw, PK 2.5: 25.III.–17.I.1986, P. Sarry leg., ex. Coll. J. Haxaire (CDH). 1 ♂, Rd. de Kaw, layon du PK 37 au km 2.6: 19.VII.2001, ex. Coll. M. Laguerre, DNA sample ID BC-Her 2708, genitalia prep. D. Herbin ref. H. 653 (CDH). 1 ♂, Nouragues research station, 4.098°N, 52.68°W: 9.IX.2010, C. Lopez Vaamonde leg., DNA sample ID BIOUG00730-A04 (MNHN). 1 ♂, Nouragues, Inselberg Camp, Heliport drop zone, 4.088°N, 52.681°W: 1.II.2011, M. Smith & R. Rougerie leg., DNA sample ID NS-RR0769 (MNHN). 1 ♂, Orapu, Crique Grillon: 13.IV.1994, P. Kindl leg. (MNHN). **GUYANA**: 1 ♂, Amazon-Courantyne divide, head of Oronoque River: 1937, H. Beddington [leg.], B.M. 1937–588 (NHMUK). 1 ♂, Potaro: II.1908, S.M. Klages [leg.], Rothschild Bequest BM 1939–1 (NHMUK). **VENEZUELA: Amazonas**: 1 ♂, Río Mavaca, 2°2'N, 65°6'W, 150 m: 16–27.III.1989, David Grimaldi leg., Exp. Phipps-Fudeci (AMNH). **BRAZIL: Amazonas**: 4 ♂, Reserva Ducke, km 26, Hwy. Manaus-Itacoatiara: 16.IV.1972, 20.IV.1972, 15.V.1972, 21.V.1972, E.G., I. & E.A. Munroe [leg.], St Laurent diss.: 5-18-16:1 (CNC). 1 ♂, Fonte Boa, Upper Amazons: VI.1906, S.M. Klages [leg.], Rothschild Bequest, BM 1939–1, NHMUK010354559, St Laurent diss.: 11-1-16:8 (NHMUK). 1 ♂, Manaus, Uypiranga, m/d [right margin] of Rio Negro: X.1941, Parko leg., N. 10.822 I. Oswaldo Cruz, USNM-Mimal: 2404 (USNM). **Pará**: 1 ♂, Ponte Nova, Rio Xingu: ex. Coll. Dognin, USNM-Mimal: 1785, St Laurent diss.: 11-1-16:4 (USNM). 1 ♂, Belém, 20 m: I.1984, V.O. Becker leg., ex. Coll. Becker 46466, USNM-Mimal: 2211 (USNM). 1 ♂, No specific locality: A.M. Moss [leg.], Rothschild Bequest, BM 1939–1 (NHMUK). **Rondônia**: 8 ♂, Porto Velho, 180 m: 24–30.IV.1989, V.O. Becker leg., ex. Coll. Becker 61968, USNM-Mimal: 2200–2207, St Laurent diss.: 11-1-16:5, 11-16:10 (USNM). 1 ♂, Vilhena, 600 m: 9.XII.1997, V.O. Becker leg., ex. Coll. Becker 111449, USNM-Mimal: 2029, St Laurent diss.: 11-16:16 (USNM). **PERU: Madre de Dios**: 1 ♂, Upper Río Madre de Dios, Manu Park, 30–40 km S Salvación, 300 m: VIII.1998 (MWM). 1 ♂, Río Madre de Diós, E. de Salvación, 300 m: VII.1998, don de Claude Lemaire (MNHN). **Huánuco**: 3 ♂, Yuyapichis, ACP Panguana, 9°36'S, 74°56'W, 220 m: VI.2013 [1 ♂], IX.2013 [2 ♂], H. Thöny leg. (MWM). 1 ♂, Yuyapichis, Fazenda Tropical, 9°37'S, 74°56'W, 210 m: VI.2013, A. Eichinger leg., Genitalia prep. No. 29.220 MWM (MWM).

#### Diagnosis.

*Reinmara enthona*, the type species of the genus *Reinmara*, is recognizable by the extensive suffusion of pinkish gray in the medial area. It is very similar to the following two species, but of the three species, *R. enthona* has the most extensive rosy medial suffusion, and a narrow submarginal area with quite falcate forewings (like *R. atlantica* sp. n., but unlike *R. andensis* sp. n.). The genitalia are intermediate in size between those of *R. andensis* and *R. atlantica*. The large diaphragmal sacks of *R. enthona* are similar to, but still smaller than those of *R. andensis*, whereas the same sacks of *R. atlantica* are about 50% smaller.

#### Description.

**Male.** *Head*: As for genus, but light brown in color. *Thorax*: Coloration as for head. *Legs*: Coloration as for thorax, vestiture thick, long. *Forewing dorsum*: Forewing length: 16–22 mm, avg.: 19 mm, wingspan: 36–43 mm, n=16. Triangular, outer margin concave below apex; tornus notched, apex usually somewhat falcate. Ground color light brown to rich chocolate brown, very sparsely scattered with tiny, dark-brown, petiolate scales. Ante- and medial areas lighter brown than darker brown submarginal area, though in some specimens medial area may be very dark brown with less suffusion of grayish pink, lighter pinkish-gray scales present throughout medial area, including near costa on outer edge of postmedial line. Antemedial line almost nonexistent. Discal spot dark ovoid mark, surrounded by pale-gray scales, darker central area variable in expanse. Fringe coloration lighter brown than wing margin. *Forewing ventrum*: Similar to dorsum but more homogenously brown overall with very obvious black splotch at costa where postmedial line meets it, covering of dark petiolate scales may be much more extensive than on dorsum. Antemedial line absent, postmedial line reduced to traces. *Hindwing dorsum*: Notch on anterior margin weak, patterning as for forewing dorsum, but antemedial line absent, discal mark and postmedial line weakly defined. *Hindwing ventrum*: Following same pattern as forewing ventrum but traces of postmedial line outwardly bent mesally. *Abdomen*: Coloration as for thorax. *Genitalia*: (Fig. 23) n=10. Typical of genus, uncus triangular but truncated distally. Gnathos with relatively short fingerlike tips of paired extensions. Valves broad, phallus somewhat conical, curved, distally quite broadened, but variable in width. **Female.** *Head*: As for male but slightly broader; antenna dentate with very small pectinations along entire length of flagellum. *Thorax*: As for male. *Legs*: As for male. *Forewing dorsum*: Forewing length: 23 mm, wingspan: 43 mm, n=1. As for male but much broader, margin convex except for just below apex; tornus strongly notched. Coloration and patterning usually as for male but medial area more uniformly pink, discal mark nearly absent. *Forewing ventrum*: Similar to dorsum but appearing browner overall due to absence of well-defined ante- and medial pink shading. Antemedial line absent, postmedial line reduced to traces, discal mark more prominent, darker than on forewing dorsum. *Hindwing dorsum*: Similar to forewing dorsum, but notch present on anterior margin, patterning as for forewing dorsum, but antemedial line absent. *Hindwing ventrum*: Following same pattern as forewing ventrum. *Abdomen*: Similar to that of male but more robust overall. *Genitalia*: (Fig. 31) n=1. Stout, robust; tergite of VIII forms elongated, posteriorly directed tongue-like overhang, VIII heavily sclerotized laterally forming curving plate below papillae anales. Apophyses anteriores roughly half-length of apophyses posteriores. Lamella ante- and postvaginalis converge as a wide, bowl-like structure covered in setae. Ductus bursae short, narrow. Balloon-like corpus bursae rather small in comparison to robust, heavily sclerotized remainder of genitalia. Papillae anales broad, apical pronounced, covered in long, fine setae.

#### Distribution

**(Fig. 36).** This species is broadly distributed throughout the Amazon rainforest at lower elevations. There are records from Venezuela, Suriname, Guyana, French Guiana, Brazil, and Peru.

#### Remarks.

Considering the expansive distribution of *R. enthona*, this name potentially includes several cryptic species. This section of the genus *Reinmara* warrants future investigation, especially on the lower and moderate elevations of the eastern Andes Mountains. We call attention to specimens from moderate elevations in Peru (MWM) and those from about 1400 m in Ecuador (MGCL) which could be *R. enthona*, *R. andensis* sp. n., or additional taxa. See remarks of *R. andensis* sp. n. for further discussion on this matter.

### Reinmara atlantica sp. n.

Taxon classificationPlantaeLepidopteraMimallonidae

http://zoobank.org/4408352E-9363-4329-AADD-EA7E0C62DD5C

Figs 8 , 24 , 36

#### Type material.

**Holotype**, ♂. **BRAZIL: Espírito Santo**: BRASIL: ES, Linhares. 40 m, 05–09.iv.1992, V.O.Becker Col/ Col. BECKER 82019/ USNM-Mimal: 2208/ St Laurent diss.: 11-1-16:6/ HOLOTYPE ♂ *Reinmara atlantica* St Laurent, Herbin, & C. Mielke, 2017 [handwritten red label]/ (ex-USNM, DZUP). Type locality: Brazil, Espírito Santo, Linhares.

#### Paratypes.

(3 ♂ total) **BRAZIL: Espírito Santo**: 2 ♂, same data and Becker number as the holotype, USNM-Mimal: 2209–2210, St Laurent diss.: 11-1-16:7 (USNM). 1 ♂, same data and Becker number as holotype (VOB).

#### Diagnosis.

*Reinmara atlantica* is very similar to *R. enthona* but is darker brown, usually slightly smaller, and has narrower forewings. Also, the light gray medial suffusions are mostly restricted to area along the postmedial line, especially near the costa, and are not present throughout the medial region as in *R. enthona*. The postmedial line is slightly angled toward the costa at Rs4 in *R. atlantica*, not interrupted there in *R. enthona*. The genitalia can be recognized by the narrower valves and smaller gnathos extensions relative to the whole of the genitalia. Perhaps the most reliable character differentiating these two species is the reduced size of all four diaphragmal sacs, especially noticeable in the lower right sac which is very reduced in comparison to that of *R. enthona*, and hardly extends inward toward the body cavity, whereas this huge sac in *R. enthona* extends well into the body cavity past the vincular ring.

#### Description.

**Male.** *Head*: As for genus, but light brown in color. *Thorax*: Coloration as for head. *Legs*: Coloration as for thorax, vestiture thick, long. *Forewing dorsum*: Forewing length: 19–20 mm, avg.: 19.7 mm, wingspan: 35–36 mm, n=3. Triangular, outer margin concave below apex; tornus notched, apex somewhat falcate. Ground color rich brown, very sparsely scattered with tiny, dark-brown, petiolate scales. Ante- and medial areas lighter brown than darker brown submarginal area, lighter gray scales present near costa on both sides of postmedial line, but more expansive on inner side with narrow strip of suffusion scales along postmedial line, fading before anterior wing margin, small patch of light-gray scales also present in antemedial area. Antemedial line almost nonexistent. Discal spot dark ovoid mark, surrounded by pale-gray scales. Fringe coloration lighter with nearly white trailing edge. *Forewing ventrum*: Similar to dorsum but more homogenously brown overall with very obvious black splotch at costa where postmedial line meets it. Antemedial line absent, postmedial reduced to traces. *Hindwing dorsum*: Notch on anterior margin weak, patterning as for forewing dorsum, but antemedial line absent, discal mark and postmedial line weakly defined. *Hindwing ventrum*: Following same pattern as forewing ventrum but traces of postmedial line outwardly bent mesally. *Abdomen*: Coloration as for thorax. *Genitalia*: (Fig. 24) n=2. Typical of genus, very similar to that of *R. enthona* but gnathos size reduced relative to whole of genitalia, diaphragm sacks much smaller overall especially lower right sac, which barely extends into body cavity past vincular ring, valves slightly narrower. **Female.** Unknown.

#### Distribution

**(Fig. 36).** *Reinmara atlantica* is known only from the type locality in Espírito Santo, Brazil near sea level in the Atlantic Forest.

#### Etymology.

This new species is named for the type locality, which is situated very near to the Atlantic coast of Brazil.

#### Remarks.

Despite an abundance of Mimallonidae material from the Brazilian Atlantic Forest in collections visited during the course of this research (see list in Methods), the four specimens from Linhares were the only *R. atlantica* material located from this hyperdiverse biome. This species may be much more restricted within this biome than other species in the family that are also endemic to the Brazilian Atlantic Forest.

### Reinmara andensis sp. n.

Taxon classificationPlantaeLepidopteraMimallonidae

http://zoobank.org/0AFC5C82-6BB5-47FB-B86F-76B76B06259C

Figs 9–11 , 25 , 36

#### Type material.

**Holotype**, ♂. **BOLIVIA**: BOLIVIE, N. Yungas, 1000–1800 m, Oct,nov,Dec,2008, Leg. local collector for R. Marx, Coll. D. Herbin/ genitalia prep. D. Herbin ref H. 1134/ HOLOTYPE male *Reinmara andensis* St Laurent, Herbin, & C. Mielke, 2017 [handwritten red label]/ (MNHN). Type locality: Bolivia, northern Yungas [no specific locality provided on data label].

#### Paratypes.

(9 ♂ total) **BOLIVIA**: 1 ♂, same data as for holotype (CDH). **La Paz**: 1 ♂, Nor [North] Yungas, Road Caranavi-Coroico, ca. 100 km NE La Paz, ca. 16.2°S, 67.6°W, 1000–1800 m: V–VI.2009, R. Brechlin & F. Meister leg. (MWM). 1 ♂, Río Songo [*recte* Río Zongo], 750 m: ex-Coll. Fassl, NHRS-TOBI 1951 (NHRS). **PERU: Puno**: 1 ♂, Santo Domingo, Carabaya, 6000 ft: I.1902, wet season, Ockenden [leg.], Rothschild Bequest, BM 1939–1, NHMUK01354562 (NHMUK). 1 ♂, Locality as for previous but: VI.1902, dry season, NHMUK 010318284 (NHMUK). 2 ♂, La Oroya [Oroya], Río Inambari, 3100 ft: III.1905, XI–XII.1905, wet season, G. Ockenden [leg.], Rothschild Bequest, BM 1939–1, NHMUK010354561, St Laurent diss.: 11-1-16:9 (NHMUK). 2 ♂, Locality and collector as for previous but: 3000 ft, V.1905, Ex-Coll. Oberthür, Brit. Mus. 1927–3, NHMUK010354560 (NHMUK).

#### Specimens of uncertain identity hereby excluded from the type series

**. ECUADOR: Napo**: 1 ♂, 1 ♀, Wildsumaco Biol. Stat., E slope Andes Mtns, 0°40'17.2"S, 77°35'55.1"W, ~1400 m: 1–14.VIII.2016, Kawahara + Barber Labs et al. leg., DNA voucher numbers LEP-40632, 42829 (MGCL, molecular collection, barcoded). **PERU: San Martín**: 1 ♂, Mina de Sal, 1400 m: V.2007, Rainer Marx leg., Genitalia prep. No. 29.219 MWM (MWM). **Huánuco**: 1 ♂, Leoncio Prado, La Divisoria, 1600 m: 20.VI.1982, Charles F. Zeiger [leg.] (MGCL).

#### Diagnosis.

*Reinmara andensis* is similar to *R. enthona* but larger, with broader wings and broader submarginal areas, which are more uniformly light brown. Medially the light gray scaling is reduced in comparison with *R. enthona*. The genitalia are very similar to those of *R. enthona*, but are overall somewhat larger, the gnathos extensions are shorter and phallus more tubular with a more protruding ventral distal lip in comparison with *R. enthona*. The lower right diaphragm sac is larger and more ovoid in shape in *R. andensis*, in *R. enthona* it is smaller and more spherical.

#### Description.

**Male.** *Head*: As for genus, but light brown in color. *Thorax*: Coloration as for head. *Legs*: Coloration as for thorax, vestiture thick, long. *Forewing dorsum*: Forewing length: 18.5–20.0 mm, avg.: 19.2 mm, wingspan: 37–40 mm, n=5. Triangular, margin slightly concave below apex; tornus notched, apex hardly falcate. Ground color light orange-brown, very sparsely scattered with tiny, dark brown, petiolate scales. Ante- and medial areas appearing lighter brown than more uniformly orange-brown submarginal area due to suffusion of lighter gray scales medially, especially near costa and on inner side of postmedial line, in some specimens medial area may be very dark brown with less suffusion of grayish pink. Antemedial line almost nonexistent. Discal mark pale gray, ovoid, variously darkened at center. Fringe coloration lighter than wing margin with nearly white trailing edge. *Forewing ventrum*: Similar to dorsum but more homogenously brown overall due to reduction in paler gray shading. Antemedial line absent, postmedial line reduced to traces. *Hindwing dorsum*: Notch on anterior margin weak, patterning as for forewing dorsum, but antemedial line absent, discal mark and postmedial line weakly defined. *Hindwing ventrum*: Following same pattern as forewing ventrum but traces of postmedial line outwardly bent mesally. *Abdomen*: Coloration as for thorax. *Genitalia*: (Fig. 25) n=4. Typical of genus, very similar to that of *R. enthona* but overall larger structures, with shorter but more robust gnathos extensions and a more tubular phallus with more prominent ventral distal lip. **Female.** Unknown [putative female from Wildsumaco, Napo, Ecuador does not differ from female *R. enthona*].

#### Distribution

**(Fig. 36).** *Reinmara andensis* is an Andean species present in southeastern Peru in the Puno region, as well as northwestern Bolivia. Other records from north central Peru in San Martín and Huánuco as well as eastern Ecuador may represent this or additional cryptic Andean taxa.

#### Etymology.

This new species is named for its Andean distribution.

#### Remarks.

Additional material from MWM and MGCL from other localities in Peru besides those from the Puno region need verification due to the unreliability of the collector and/or unclear collecting data. We anticipate that this new species is more broadly distributed, but considering the close similarity to *R. enthona* and unavailability of recently collected Peruvian material, we restrict the type series of this species to include only those from northwestern Bolivia and adjacent southeast Peru. Although *R. andensis* is endemic to the eastern slopes of the Andes, it appears to be sympatric with *R. enthona* at the lower elevations in the inhabited range of *R. andensis*.

Due to the barcoding results (Fig. 7) and biogeography placing an Ecuadorian specimen (Lep-40632) closer to *R. andensis*, we have included specimens from this location under additional examined material for *R. andensis*, though they are excluded from the type series pending additional information. Furthermore, these barcoding results are not clear in that *R. wolfei* (Bc-Her4822) is nested within the clade including *R. andensis* and the Ecuadorian R. cf andensis, with low bootstrap support. Morphology certainly suggests that *R. enthona* and *R. andensis* are more similar than the rather unique, *R. wolfei*. Additional molecular and morphological data will be required to fully elucidate the relationships within *Reinmara*. We do not consider single genes, particularly COI, to offer significant phylogenetic signal, especially considering recent work refuting species delimitation based on genetic evidence alone (Sukumaran and Knowles 2017), thus we include the tree in Fig. 7 merely as additional evidence differentiating the Amazonian *R. enthona* from the externally similar Andean *R. andensis*.

In the NHMUK, the Peruvian specimens were collected both during the “dry season” and “wet season” with those specimens from the dry season being smaller overall than those from the wet season. No significant genitalia differences were noted between these sets of specimens however. *Reinmara andensis* is generally larger than *R. enthona* but dry season *R. andensis* are much closer in size to those of *R. enthona*.

### Reinmara occidentalis sp. n.

Taxon classificationPlantaeLepidopteraMimallonidae

http://zoobank.org/2A610073-44B4-48BD-BF4A-F0808DA6530B

Figs 12 , 26 , 27 , 36

Psychocampa nocturna ‡ in Piñas 2007, fig. 215 ♂, ***nomen nudum***

#### Type material.

**Holotype**, ♂. **ECUADOR: El Oro**: ECUADOR, El Oro prov. 10km NW PIÑAS, 3°38'51"S, 79°45'52"W, 12.04.2012; H=750 m, leg. R. Brechlin & V. Sinyaev, Museum Witt/ Genitalpräparat Heterocera Nr. 29.218 Musuem WITT München/ HOLOTYPE male *Reinmara occidentalis* St Laurent, Herbin, & C. Mielke, 2017 [handwritten red label]/ (MWM). Type locality: Ecuador, El Oro, 10 km NW of Piñas.

#### Paratype.

**ECUADOR: El Oro**: 1 ♂, Road Piñas-Saracay, 3°39'52"S, 79°45'26"W, 800 m: 6.XII.2012, Sinyaev & Romanov, expedition Ron Brechlin leg., genitalia prep. 30.813 (MWM).

#### Diagnosis.

*Reinmara occidentalis* is one of most obscurely colored species in the genus. This new species is recognizable by the lack of a well-defined notch on the forewing tornus, which is instead smooth, and by the dark brown submarginal coloration with an almost complete absence of gray/pink shading in the medial region. On the ventral surface of the wings, the postmedial line is more continuous and less intermittently notched than in *R. enthona*, *R. atlantica*, or *R. andensis*. The male genitalia are also unique in this species because the gnathos extensions are quite long and deeply divergent, and the phallus is somewhat twisted, noticeably bent, and broadened distally unlike any other in the genus. This species is so far the only *Reinmara* known from the western slopes of the Andes.

#### Description.

**Male.** *Head*: As for genus, but dark brown in color. *Thorax*: Coloration as for head but slightly lighter brown. *Legs*: Coloration as for thorax, vestiture thick, long. *Forewing dorsum*: Forewing length: 22.5–23.5 mm, avg.: 23 mm, wingspan: 40–42 mm, n=2. Triangular, outer margin weakly concave below apex; tornus smooth, unnotched, apex somewhat falcate. Ground color brown, sparsely scattered with dark brown, tiny petiolate scales. Ante- and medial areas lighter brown than darker, chocolate brown submarginal area, lighter gray scales present near costa on both sides of postmedial line. Antemedial line light brown but darker than surrounding area, wavy. Discal mark ovoid, surrounded by pale gray scales. Fringe coloration lighter brown than submarginal area. *Forewing ventrum*: Similar to dorsum but more homogenously brown overall, pale gray shading more evident near apex and submarginally. Antemedial line absent, postmedial line as on dorsum but fainter. *Hindwing dorsum*: Anterior margin without notch, but edge flatter than mesal wing margin. Patterning as for forewing dorsum, but antemedial line absent, discal mark and postmedial line weakly defined. *Hindwing ventrum*: Following same pattern as forewing ventrum but postmedial line outwardly bent mesally. *Abdomen*: Coloration as for thorax. *Genitalia*: (Fig. 26, 27) n=2. Typical of genus, differing in the more robust gnathos mesal extensions with particularly elongated fingerlike tips, phallus twisted, bent mesally, and distally broadened. **Female.** Unknown.

#### Distribution

**(Fig. 36).** *Reinmara occidentalis* is known from only two locations separated by a little over 2 km in the El Oro province of western Ecuador, on the western slopes of the Andes mountains from 750–800 m in elevation.

#### Etymology.

This new species is named for the western (*occidentalis* Latin) Andean distribution.

#### Remarks.

We are only aware of two specimens of this new species. Although data is still lacking in regards to the extent of the distribution of *R. occidentalis*, the distribution as well as the external morphology of this species are quite distinct from all others in the genus.

A specimen that may represent this new species was figured (fig. 215) in the plates of Piñas (2007) with the unavailable name *Psychocampa nocturna*‡ Piñas assigned by the author. As per information available in Thöny and Piñas (2015, 2017), all names proposed by Piñas in his works “Mariposas del Ecuador” are unavailable and must be regarded as *nomina nuda* since they do not satisfy ICZN requirements for taxonomically available name (e. g. no description is provided). Thus, we above treat this name as *nomen nudum*. While the specimen figured in Piñas (2007) closely resembles *R. occidentalis* by the obscured coloration, there is a weak notch present at the tornus of the forewings, thus we cannot say for certain if it is indeed this species. Furthermore, locality information is not available, so we are not able to verify if the locality for this particular specimen satisfies our understanding of the west Andean distribution of *R. occidentalis*. The listed wingspan of 44 mm is greater than that of either specimen that we have examined.

### Reinmara wolfei

Taxon classificationPlantaeLepidopteraMimallonidae

Herbin & C. Mielke, 2014

Figs 13–16 , 28 , 32 , 36

Reinmara wolfei Herbin and Mielke, 2014: 144, figs ♂ 40, 41, 43

#### Type material.

**Holotype**, ♂. **BRAZIL: Maranhão**: holotype, *Reinmara wolfei* HERBIN & MIELKE det./ Brésil, Maranhão, Feira Nova do Maranhão, Retiro, 480 m, 24/31-XII-2011, 07°00'31"S, 46°26'41"W, C. MIELKE leg./ DZ 15.713/ Genitalia prep. D. Herbin ref. H 953/ (DZUP, examined). Type locality: Brazil, MA, Feira Nova do Maranhão.

#### Additional material examined.

(7 ♂, 4 ♀ total) **BRAZIL: Maranhão**: 1 ♂, Balsas, 8°38'S, 46°43'W, 525 m: Coll. EMBRAPA-CPAC No. 20907 (CPAC). **Goiás**: 1 ♂, 2 ♀, Leop. Bulhoes [Leopoldo de Bulhões]: XI.1935, III.1936, ex. coll. R. Spitz, H.R.P[earson] genitalia prep. 4184 [lost], NHMUK010354557, 010354558 (2 ♀, NHMUK); XII.1936, ex. coll. R. Spitz, HRP No. 1462 (1 ♂, MNRJ). **Distrito Federal**: 1 ♂, Brasília: 25.II.1966, ex. coll. Gagarin (DZUP). 2 ♂, 2 ♀, Planaltina, 15°35'S, 47°42'W, 1000 m: 11.XI.1976, 31.III.1977, 9.III.1978, 4.IV.1978, V.O. Becker leg., Coll. EMBRAPA-CPAC No. 2425, 4940, 6812, 6879 (CPAC). **Mato Grosso**: 1 ♂, 60 km south of Poconé, Pantanal, 100 m: 22.V.1998, V.O. Becker leg., ex. Coll. Becker 116547, St Laurent diss.: 11-1-16:2 (USNM). **BOLIVIA: Santa Cruz**: 1 ♂, Aguas Calientes [Roboré]: Travassos, Barros & Albuquerque leg. (CEIOC).

#### Diagnosis.

*Reinmara wolfei* is characterized by the small size, sandy, tan brown coloration, only very faint to absent paler shading medially, and a faint or absent discal mark on the hindwing ventrum. The phallus of *R. wolfei* is the shortest and broadest of the genus. The female genitalia are not overly distinct from those of *R. enthona*.

#### Description.

**Male.** *Head*: As for genus, coloration brown, antenna coloration brown. *Thorax*: Coloration lighter brown than that of head. *Legs*: Coloration as for thorax. *Forewing dorsum*: Forewing length: 15–17 mm, avg.: 16.3 mm, wingspan: 30–36 mm, n=4. Triangular, outer margin concave, tornus weakly notched. Ground color sandy brown. Ante- and medial areas concolorous, submarginal area above tornus slightly darker brown than remainder of wing in fresh specimens, pale suffusion present on inner side of postmedial line near costa. Antemedial line faint brown, wavy, postmedial line slightly curved, usually thick, black. Discal mark weakly represented by pale splotch with darkened region centrally. Fringe coloration as for remainder of wing or slightly darker. *Forewing ventrum*: Similar to dorsum but pale suffusions most absent except near apex. Antemedial line absent, postmedial line reduced to wavy traces, discal mark more prominent, darker than on forewing dorsum. *Hindwing dorsum*: Notch on anterior margin weak, patterning as for forewing dorsum, but antemedial line absent, discal mark and postmedial line weakly defined. *Hindwing ventrum*: Following same pattern as forewing ventrum. *Abdomen*: Coloration as for thorax. *Genitalia*: (Fig. 28) n= 4. Typical of genus, differing in the relatively triangular shape of the uncus, more elongated gnathos mesal extensions with particularly elongated fingerlike tips that are usually slightly bent, sacculus fold with large tooth-like extensions, phallus short, blunt, broad, covered in fine setae. **Female.** *Head*: As for male but slightly broader; antenna dentate with very small pectinations along entire length of flagellum. *Thorax*: As for male. *Legs*: As for male. *Forewing dorsum*: Forewing length: 15–19 mm, avg.: 17.3 mm, wingspan: 33–35 mm, n=4. As for male but much broader, margin convex except for just below apex; tornus strongly notched. Coloration and patterning as for male but discal mark almost entirely absent. *Forewing ventrum*: Similar to dorsum but more homogenously brown overall due to absence of well-defined ante- and medial areas. Antemedial line absent, postmedial line reduced to outwardly curved traces, discal mark more prominent, discal mark darker than on forewing dorsum. *Hindwing dorsum*: Similar to forewing dorsum, but notch present on anterior margin, patterning as for forewing dorsum, but antemedial line absent, discal mark and postmedial line usually weakly defined. *Hindwing ventrum*: Following same pattern as forewing ventrum. *Abdomen*: Similar to that of males but more robust overall. *Genitalia*: (Fig. 32) n=1. Stout, robust; tergite VIII forms elongated, posteriorly directed shortened tongue-like overhang, VIII heavily sclerotized laterally forming curving plate below papillae anales. Apophyses anteriores roughly half-length of apophyses posteriores. Lamella ante- and postvaginalis converge as a wide, bowl-like structure covered in setae. Ductus bursae short, narrow. Corpus bursae rather small in comparison to robust, heavily sclerotized remainder of genitalia, balloon-like. Papillae anales broad, apical pronounced, covered in long, fine setae.

#### Distribution

**(Fig. 36).** *Reinmara wolfei* is endemic to the Cerrado of central South America, with few records from Brazil in the states of Maranhão, Goiás, and Distrito Federal. We also report a specimen from the wet Pantanal in Brazil, Mato Grosso. A specimen from Cerrado habitat in Bolivia, Santa Cruz, is reported here as well.

#### Remarks.

We figure and describe the female of this species for the first time, as well as the first Bolivian record. Until now, this species was known only from the male holotype from Maranhão, Brazil. We note some minor external differences between the specimens from drier Cerrado and that of the wet Pantanal, such as the slightly smaller size and brighter coloration in the Pantanal specimen (Fig. 15), but genitalia of this specimen are not noticeably different from those of typical *R. wolfei*.

### Reinmara minasa

Taxon classificationPlantaeLepidopteraMimallonidae

Schaus, 1928

Figs 17–19 , 29 , 33 , 36

Reinmara minasa Schaus, 1928: 655, fig. ♂ 88b
Reinmara minasa ; Gaede 1931
Reinmara minasa ; Becker 1996
Reinmara minasa ; Herbin and Mielke, 2014

#### Type material.

**Holotype**, ♂. **BRAZIL: Minas Gerais**: Passa Quatro, Sul de Minas [SE of Minas Gerais], S.O. Brasilien, Jos. Zikán [leg.]/ [Holo]Typus/ No. [illegible] 6, 19-I-22/ *Reinmara minasa* Schaus type/ (MNHU, examined). Type locality: Brazil, Minas Gerais, Passa Quatro.

#### Additional specimens examined.

(39 ♂, 2 ♀ total) **BRAZIL: Espírito Santo**: 1 ♂, No additional data, St Laurent diss.: 5-15-16:1 (CUIC). **Minas Gerais**: 1 ♂, Itamonte, Vargem Grande, 1600 m: 17.II.2010, [O.] Mielke & Casagrande leg. (DZUP). 1 ♂, Alto Caparaó, Tronqueira, 20°24'38"S, 41°50'07"W, 1994 m: 10.XI.2012, B. Vincent leg., BC-Her4979, genitalia prep. D. Herbin ref. H. 1132 (CDH). **Rio de Janeiro**: 3 ♂, [Itatiaia], Pico de Itatiaia: 28.III–1.IV.1958, H.B.D. Kettlewell [leg.], B.M. 1958–273 (NHMUK). 3 ♂, Itatiaia, L. 41, 1300 m: 3–8.II.1951, Trav[assos] & Albuquerque [leg.] (NHMUK, 2 ♂); 6–10.XII.1950, 270, USNM-Mimal: 2422, St Laurent diss.: 11-1-16:1 (1 ♂, USNM). 1 ♂, Itatiaia, 700 m: 3.IV.1927, J. Zikán leg., ex-Coll. Gagarin (DZUP). 1 ♂, Itatiaia, 1200 m: II.1960, H. Ebert leg. (ZSM). 2 ♂, Parque Nacional do Itatiaia, Lago Azul, 800 m: 19.III.1955, G. & H. Pearson leg., HRP No. 784, USNM-Mimal: 2381 (USNM); 14–17.IV.1956, Pearson & R. Barros [leg.], HRP No. 776, USNM-Mimal: 2382 (USNM). 1 ♂, [Itatiaia], Campo Bello [Campo Belo]: Zikán leg., USNM-Mimal: 1788 (USNM). **São Paulo**: 7 ♂, 1 ♀, Campos do Jordão [Santo Antônio do Pinhal], Eugênio Lefèvre, 1200 m: 13–20.XI.1952, L. Travassos Filho, D’Almeida, & Pd. Pereira [leg.]; 15–20.XII.1952, L. Travassos Filho & D’Almeida [leg.]; 14–17.I.1953, L. Travassos Filho & S. Medeiros [leg.]; 13–15.II.1953, L. Travassos Filho & L. Travassos [leg.]; 22.III.1963, L. Travassos Filho, J. Guimarães, E. Rabello, & A. Barroso [leg.], MSZP Nos. 28065–28071, ♀ genitalia prep. MZSP 28071 (6 ♂, 1 ♀, MZSP); 16.XII.1952, D’Almeida & L. Travassos F. leg., Ex-coll. D’Almeida (1 ♂, DZUP). 1 ♂, Eugênio Lefèvre [train station, Santo Antônio do Pinhal], 1162 m: ex. Coll. Gagarin (DZUP). 2 ♂, Campos do Jordão, Umuarama, 1800 m: 3–15.II.1937 [DZ 33.014], 8–15.III.1937, Gagarin leg., ex. Coll. Gagarin (DZUP). 6 ♂, São José do Barreiro, Bocaina, 44°37'57"W, 22°43'37"S, 1539 m: 2–6.I.2016, C. Mielke leg., CGCM 31.240, CGCM 31.263, CGCM 31.274, CGCM 31.285, CGCM 31.310, CGCM 31.331 (CGCM). 1 ♂, São José do Barreiro, Bocaina, 44°39'49"W, 22°44'35"S, 1692 m: 9–10.X.2015, C. Mielke leg., CGCM 30.813 (CGCM). 1 ♂, Termas de Lindóia [Águas de Lindóia]: 27.I.1950, N. & R. D’Almeida leg., ex. Coll. D’Almeida (DZUP). 1 ♂, Anhembi, Faz. Bar. Rico: 1.III.1960, LTF A. Barroso (MZSP). 1 ♀, Termas de Lindoia [*recte* Aguas de Lindóia]: 10.II.1950, D’Almeida leg. (MNRJ). **Paraná**: 3 ♂, [Piraquara], Banhados, railroad from Curitiba to Paranaguá, 800 m: 11.II.1972, E.G., I. & E.A. Munroe [leg.], St Laurent diss.: 5-8-16:2 (CNC). 2 ♂, Tibagi, Guartelá, 975 m: 18.I.2012, 3.III.2012, C. Mielke leg. (CDH). **Santa Catarina**: 1 ♂, Serra do Panelão, Urubici, 27°53.989'S, 49°35.156'W, 1250 m: 26–27.II.2007 (CDH).

#### Diagnosis.

This unique species of *Reinmara* can be recognized by the black suffusion along the entire length of the forewing postmedial line in males, which reaches the apex, darkening it. In both males and females there is a well-defined, narrow, pale pink suffusion along the postmedial line (outside of the black suffusion of the males, which is absent in females), leaving the remainder of the medial area mostly clear of pale pink suffusions. The male genitalia is recognizable by the uniformly narrow phallus with a usually distinctly backward splayed distal ventral tip, the uncus is quite broad. Among the species for which the female is known, *R. minasa* female genitalia is characterized by the largest dorsal projection of the tergite VIII as well as by the robustness of the lateral plates below the papillae anales.

#### Description.

**Male.** *Head*: As for genus, coloration light brown. *Thorax*: Coloration as for head but with pale pink scales present on prothoracic collar and base of wings. *Legs*: Coloration as for thorax, but with additional, dark petiolate scales sparsely scattered amongst vestiture, tarsus yellower. *Forewing dorsum*: Forewing length: 16.5–21.0 mm, avg.: 18.1 mm, wingspan: 33.0–42.5 mm, n=9. Acutely triangular, narrow, outer margin concave; tornus deeply notched nearly until postmedial line, apex somewhat falcate. Ground color brown, very sparsely scattered with dark brown, tiny petiolate scales. Antemedial area with pale pink hue, medial area displaying narrow strip of ground color between pink hue of antemedial area and inner pink suffusion of postmedial line, submarginal area darker brown than medial area with pale gray lunule-like marking on margin and strong, black suffusion on outer edge of postmedial line, black suffusion becoming widest and more diffuse near tornus, extending along entire postmedial line to apex. Antemedial line hardly distinguishable but present as outwardly bent brown wave, postmedial line nearly straight. Discal mark variable from pale pink splotch with little to no black scales in center to almost entirely covered by black scales. Fringe coloration nearly white with darker scales at wing vein intersections. *Forewing ventrum*: As for genus but pale pink scales along postmedial line broadly scattered, postmedial line as on dorsum straight, but only fainter, black suffusion replaces lunule-like submarginal shape of dorsum. *Hindwing dorsum*: Notch on anterior margin weak, patterning as for forewing dorsum, but antemedial line absent, discal mark nearly always absent, pale suffusion submarginally similar to forewing lunule-like area. *Hindwing ventrum*: Following same pattern as forewing ventrum but postmedial line wavier, discal mark present, pale pink suffusion widely expanded throughout medial and submarginal areas. *Abdomen*: As for genus. *Genitalia*: (Fig. 29) n=4. Typical of genus, differing in the relative shortness and (usual) broadness of uncus, generally more robust gnathos mesal extensions with particularly elongated fingerlike tips, phallus narrow and smoothly curved, somewhat boomerang shaped, tip of phallus splayed open with ventral edge forming backwardly angled lip. Vesica bulbous with distally extended narrower portion. **Female.** *Head*: As for male, but antenna dentate with very small pectinations along entire length of flagellum. *Thorax*: As for male. *Legs*: As for male. *Forewing dorsum*: Forewing length: 21 mm, wingspan: 43 mm, n=1. As for male but much broader, margin nearly straight. Coloration and patterning as for male except outer black suffusion along postmedial line absent. *Forewing ventrum*: Similar to dorsum but lighter, homogenous brown without distinctly different areas of wing except for darker brown region submarginally. Antemedial line absent, postmedial line very faint, discal mark more prominent, darker than on forewing dorsum. *Hindwing dorsum*: Similar to forewing dorsum, notch present on anterior margin, patterning as for forewing dorsum, but antemedial line and discal mark absent. *Hindwing ventrum*: Following same pattern as forewing ventrum. *Abdomen*: Similar to that of males but more robust overall. *Genitalia*: (Fig. 33) n=1. Very stout, robust; tergite of VIII forms elongated, posteriorly directed tongue-like overhang, VIII heavily sclerotized laterally forming curving plate encircling the papillae anales, curved plate weakly curling backward near papillae anales. Apophyses anteriores roughly half-length of apophyses posteriores. Lamella ante- and postvaginalis converge as a wide, bowl-like structure covered in setae. Ductus bursae short, narrow. Corpus bursae rather small in comparison to robust, heavily sclerotized remainder of genitalia, baglike. Papillae anales broad, rounded, covered in long, fine setae.

#### Distribution

**(Fig. 36).** *Reinmara minasa* is endemic to southeastern to south Brazil, and is found in mountainous regions of the states of Espírito Santo, Minas Gerais, Rio de Janeiro, São Paulo, Paraná, and Santa Catarina, at elevations ranging from 700–2000 m.

#### Remarks.

Until now, very little has been reported on this species. We figure and describe the female of *R. minasa* for the first time.

### Reinmara ignea sp. n.

Taxon classificationPlantaeLepidopteraMimallonidae

http://zoobank.org/60EF2888-A2B1-43E5-AA6D-3D40BAF74821

Figs 20–22 , 30 , 34–35 , 36

#### Type material.

**Holotype**, ♀. **BRAZIL: Santa Catarina**: BRAZIL – SC, São Bento do Sul, Rio Natal, 550 m., (no date). I. Rank leg./ 20.982 Col. C. Mielke [dissection number equivalent]/ HOLOTYPE female *Reinmara ignea* St Laurent, Herbin, C. Mielke, 2017 [handwritten red label]/ (DZUP). Type locality: Brazil: Santa Catarina: São Bento do Sul, Rio Natal.

#### Paratypes.

(1 ♂, 1 ♀ total) **BRAZIL: Santa Catarina**: 1 ♂, São Bento do Sul, Rio Vermelho, 968 m: 26.II.1973, A. & J. Razowski leg., St Laurent diss.: 5-6-16:1 (ISEZ). **Rio de Janeiro**: 1 ♀, Nova Friburgo, 1100 m: 21.I.1998, V.O. Becker leg., ex. Coll. Becker 112810, St Laurent diss.: 2-29-16:1 (USNM).

#### Diagnosis.

This unique species cannot be confused with any other Mimallonidae. *Reinmara ignea* is the smallest species of *Reinmara*, bearing little outward resemblance to others of the genus. The tiny size, sharply acute and falcate forewings, thick postmedial and antemedial lines, narrow and curving phallus, are just the most immediately recognizable characters enabling the identification of this new species. We also note that this is the only species of *Reinmara* for which the female has bipectinate antennae like the male (albeit smaller overall), not dentate as in other female *Reinmara*.

#### Description.

**Male.** *Head*: As for genus but coloration pale beige, antenna coloration pale brown due to scaling, but much darker brown beneath scales, vestigial proboscis not visible. *Thorax*: Coloration as for head. *Legs*: Coloration as for thorax, vestiture homogenously colored. *Forewing dorsum*: Forewing length: 12 mm, wingspan: 24 mm, n=1. Triangular, outer margin concave; tornus weakly notched, apex falcate. Ground color light orange-brown, speckling of tiny petiolate scales. Ante- and medial areas concolorous, darker brown than submarginal area, submarginal area much lighter orange-brown, appearing nearly yellow, faint pale lunule-like marking along margin below apex. Antemedial line defined, dark brown, slightly outwardly bowed, postmedial line also dark brown, slightly wider than antemedial line, barely curved. Discal mark as pale splotch, with obscured, darker central region. Fringe not well preserved. *Forewing ventrum*: Compared to forewing dorsum, more subdued tan brown, homogenous across all areas of wing, antemedial line absent, postmedial line as for dorsum, petiolate scaling heavier, especially antemedially, discal mark dark brown streak. *Hindwing dorsum*: Shape more rounded than forewing, outer margin convex except straight anterior margin, patterning as for forewing dorsum but both ante- and medial areas lighter, more similar to submarginal area in coloration, antemedial line absent, postmedial line as for forewing dorsum, well defined, discal mark present but weakly as pale streak. *Hindwing ventrum*: Following same pattern as forewing ventrum. *Abdomen*: As for genus. *Genitalia*: (Fig. 30) n= 1. Rather typical of genus, differing in smaller setae-filled sacks in diaphragm, which contain fewer setae, a more triangular, truncated uncus, gnathos round rather than rectangular, with triangular, dual mesal extensions that are fused together, extensions barely separated distally into short paired, fingerlike tips, sacculus fold particularly well developed and more symmetrical, phallus strongly curved, distally flattened and bent. **Female.** *Head*: As for male but slightly darker in color; antenna bipectinate and similar to that of male, but slightly smaller overall. *Thorax*: As for male but darker brown. *Legs*: As for male but darker brown overall with lighter yellow tarsus, tibial spurs more heavily clothed in scales. *Forewing dorsum*: Forewing length: 12–16 mm, avg.: 14 mm, wingspan: 27–31 mm, n=2. Shape essentially as for male but tornus slightly notched. Maculation as for male, but coloration darker orange-brown to red brown submarginally. Submarginal area proportionally wider. *Forewing ventrum*: Compared to forewing dorsum, more subdued tan brown, homogenous across all areas of wing, antemedial line absent, postmedial line as for dorsum, petiolate scaling heavier, especially antemedially, discal mark dark brown streak. *Hindwing dorsum*: As for male but medial and submarginal areas more distinctly bicolored (similar to forewing dorsum). *Hindwing ventrum*: Following same pattern as forewing ventrum. Frenulum as multiple bristles. *Abdomen*: Similar to that of male but more robust overall. *Genitalia*: (Figs 34, 35) n=2. Tergite of VIII forming short, thin posteriorly directed extension, VIII sclerotized laterally forming curving plate, but not extended to encircle papillae anales. Apophyses anteriores roughly equal in length apophyses posteriores. Lamella ante- and postvaginalis converge as a wide, bowl-like structure. Ductus bursae short, rectangular. Corpus bursae elongate, tubular. Papillae anales somewhat narrow, covered in long, fine setae.

#### Distribution

**(Fig. 36).** *Reinmara ignea* is so far known only from two nearby localities in São Bento do Sul, Santa Catarina, and a third locality in Rio de Janeiro State, Brazil. These two areas are separated by about 815 km and both fall in the mountainous region of the Brazilian Atlantic Forest.

#### Etymology.

This new species is named for its fiery (*ignea* Latin) coloration, reminiscent of burning embers.

#### Remarks.

Until the first author dissected the single male of this new species, proper generic placement was not clear to us, and we had originally considered *R. ignea* as belonging to an undescribed genus. Despite the outward uniqueness of both sexes, the genitalia of both sexes display characters fundamental to the diagnosis of the genus *Reinmara*. In the male, the structure and shape of the valves, the broad, mesally fused but distally separated gnathos, and balloon-like setae-filled sacs extending inward into the body cavity from the diaphragm are all typical of *Reinmara*, the gnathos character precluding *R. ignea* from placement in the related *Trogoptera*. Female genitalia are similar to those of other species of *Reinmara*, but the tergite VIII extension is particularly weakly sclerotized and thin (though present). We also note that this is the only species in the genus for which the female antennae are similar (bipectinate) to those of the male, just smaller, as in most mimallonid genera, not dentate as in the females of *R. enthona*, *R. wolfei*, and *R. minasa*.

We note minor difference in maculation of the two female specimens of *R. ignea* (compare Figs 21 and 22), as well as in their genitalia, but due to the otherwise close similarity (in comparison with other species in the genus) and the apparent rarity of this species, we include both specimens in the type species.

This species and *R. atlantica* may very well be of conservation concern due to the present state of fragmentation of the biome to which they are endemic (Ribeiro et al. 2009). The lack of specimens of *R. ignea* from this otherwise relatively well-collected region suggests that it may be rare and/or only weakly attracted to light. It is notable that most specimens of *R. ignea* are female. The opposite is true for other *Reinmara* where both sexes are known, where males far outnumber collected females.

Two additional female specimens were located in the collection of Ivo Rank, collector of the holotype, but they are not included in the type series.

## 

**Figures 1–5.**
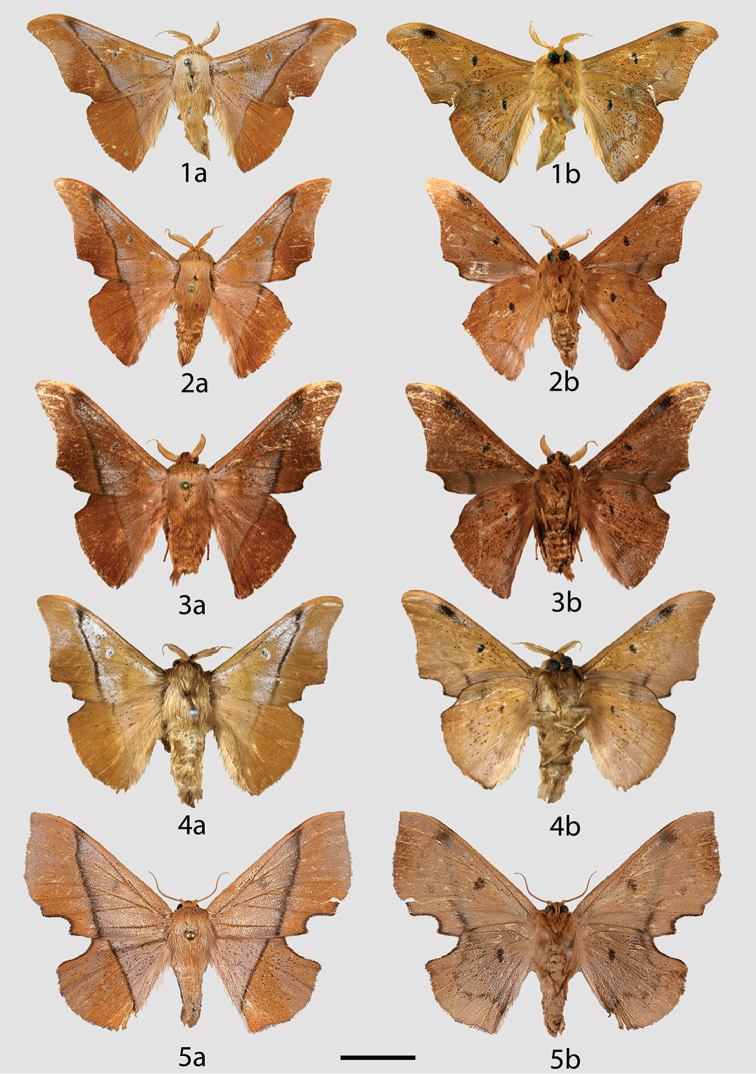
*Reinmara enthona* adults, **a** dorsal **b** ventral. **1** Holotype ♂, French Guiana, St. Jean du Maroni (USNM) **2** ♂, French Guiana, Kaw Rd., Camp Patawa (CDH) **3** ♂, French Guiana, Patagai Rd., 58 m (CDH) **4** ♂, Brazil, Rondônia, Porto Velho, 180 m (USNM) **5** ♀, French Guiana, Kaw Rd., PK 37.5 + 2, 200 m (CDH). Scale bar: 1 cm.

**Figure 6.**
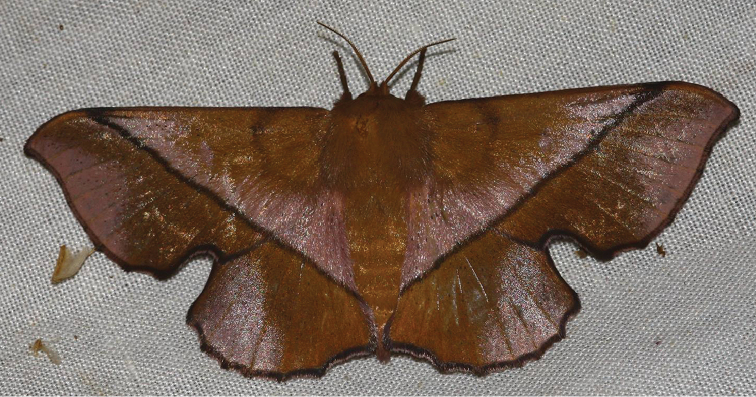
*Reinmara enthona* ♀, Suriname, Brokopondo, Brownsberg NP, 490 m (Photograph courtesy of A.J. Hielkema, used with permission).

**Figure 7.**
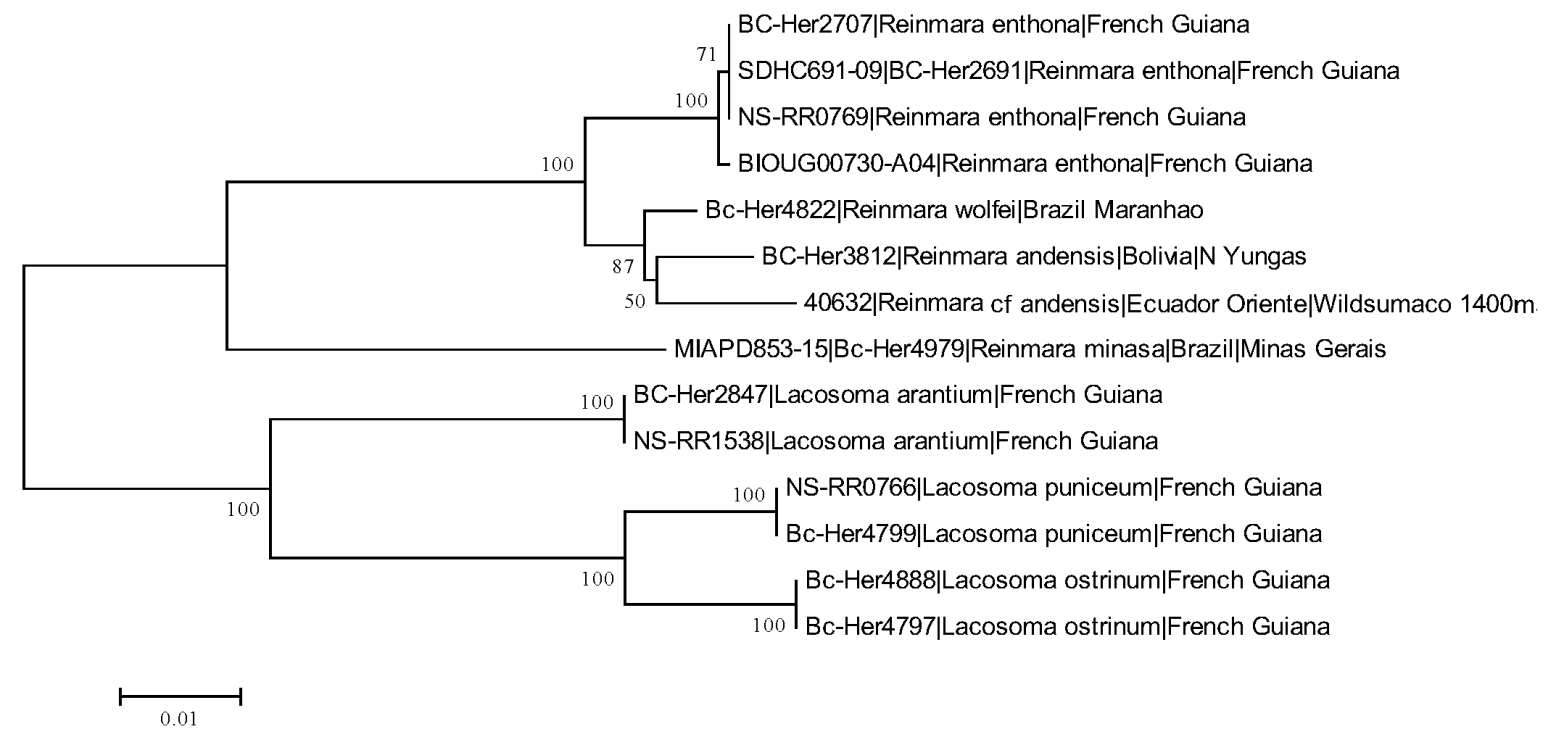
Phylogenetic tree built with neighbor-joining method in MEGA6 showing relationships among *Reinmara*, with Lacosoma Grote, 1864 as the outgroup. See remarks section for R. andensis regarding noted issues with this analysis.

**Figures 8–12.**
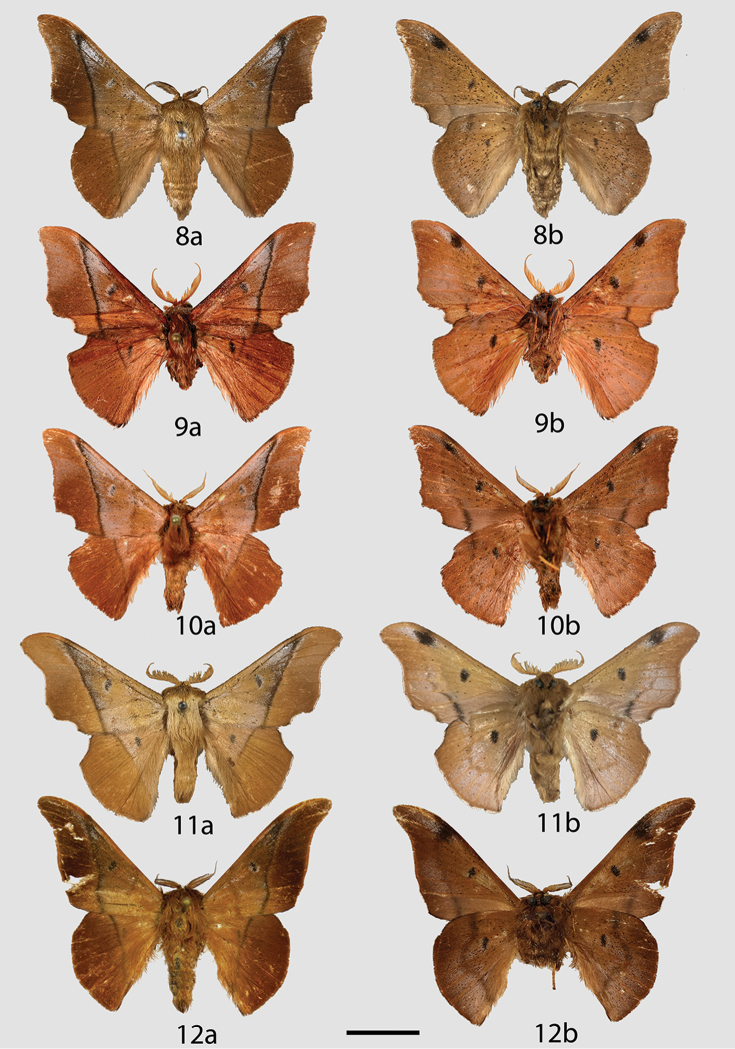
*Reinmara* adults, **a** dorsal **b** ventral. **8** *R. atlantica* Holotype ♂, Brazil, Espírito Santo, Linhares, 40 m (DZUP) **9** *R. andensis* Holotype ♂, Bolivia, N. Yungas, 1000–1800 m (MNHN) **10** *R. andensis* Paratype ♂, Locality as for Fig. 9 (CDH) **11** *R. andensis* Paratype ♂, Peru, Puno, Oroya, Río Inambari, 3100 ft (NHMUK) **12** *R. occidentalis* Holotype ♂, Ecuador, El Oro, 10 km NW Piñas, 750 m (MWM). Scale bar: 1 cm.

**Figures 13–16.**
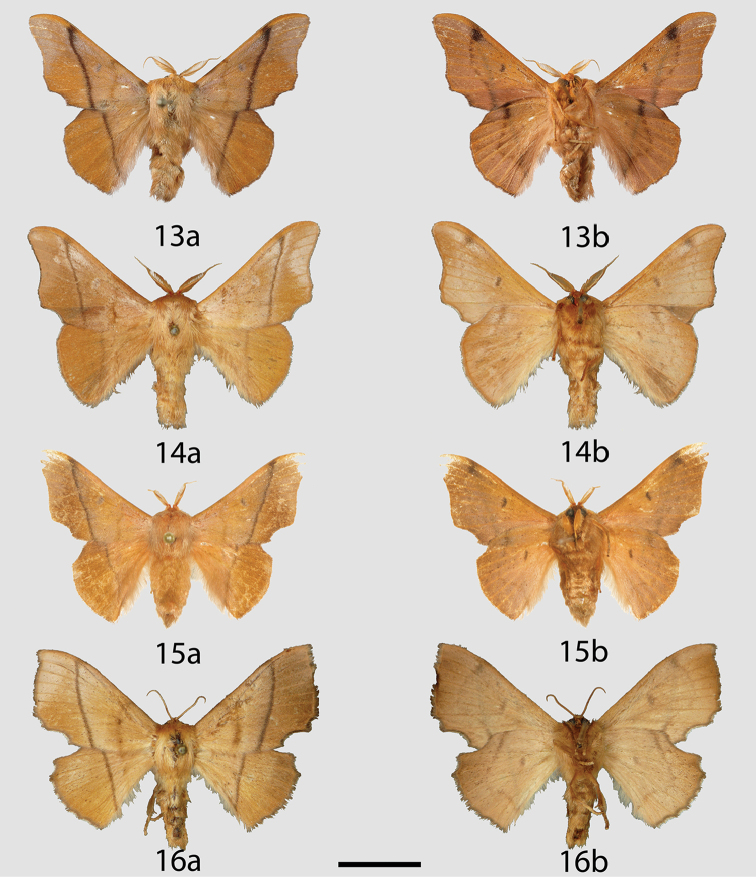
*Reinmara wolfei* adults, **a** dorsal **b** ventral. **13** Holotype ♂, Brazil, Maranhão, Feira Nova do Maranhão, 480 m [image originally published by Antenor, reused with permission] (DZUP) **14** ♂, Brazil, Distrito Federal, Planaltina, 1000 m (CPAC) **15** ♂, Brazil, Mato Grasso, 60 km S. of Poconé, Pantanal, 100 m (USNM) **16** ♀, Locality as for Fig. 14 (CPAC). Scale bar: 1 cm.

**Figures 17–22.**
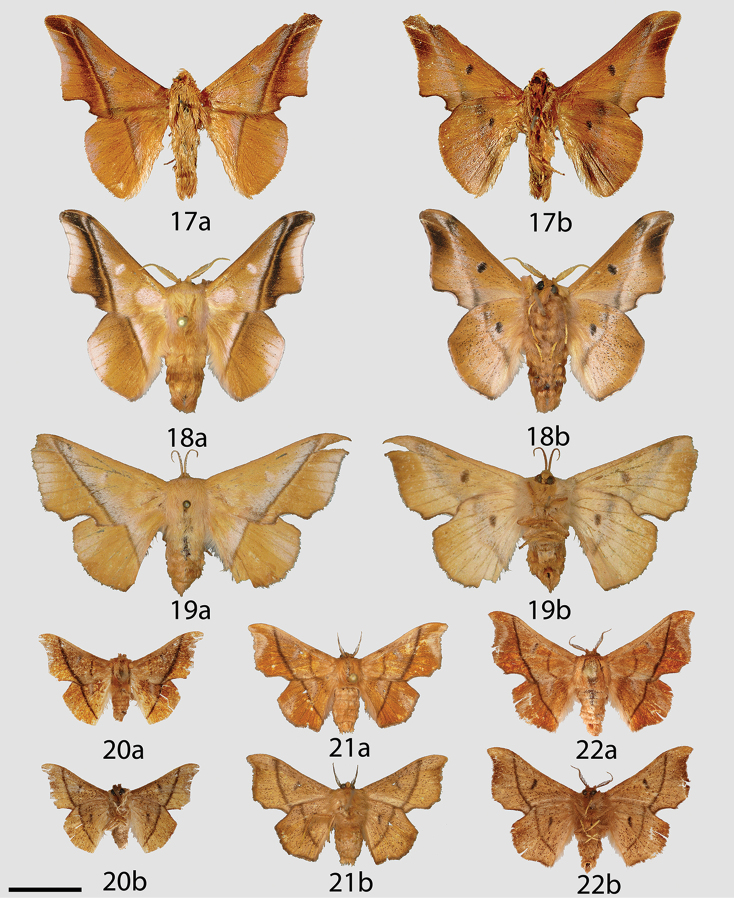
*Reinmara* adults, **a** dorsal **b** ventral. **17** *R. minasa* Holotype ♂, Brazil, Minas Gerais, Passa Quatro (MNHU) **18** *R. minasa* ♂, Brazil, São Paulo, São José do Barreiro, Bocaina, 1539 m (CGCM) **19** *R. minasa* ♀, Brazil, São Paulo, Santo Antônio do Pinhal, Eugênio Lefèvre, 1200 m (MZSP) **20** *R. ignea* Paratype ♂, Brazil, Santa Catarina, Rio Vermelho, 968 m (ISEZ) **21** *R. ignea* Holotype ♀, Brazil, São Bento do Sul, Rio Natal, 550 m (DZUP) **22** *R. ignea* Paratype ♀, Brazil, Rio de Janeiro, Nova Friburgo, 1100 m (USNM). Scale bar: 1 cm.

**Figures 23, 24.**
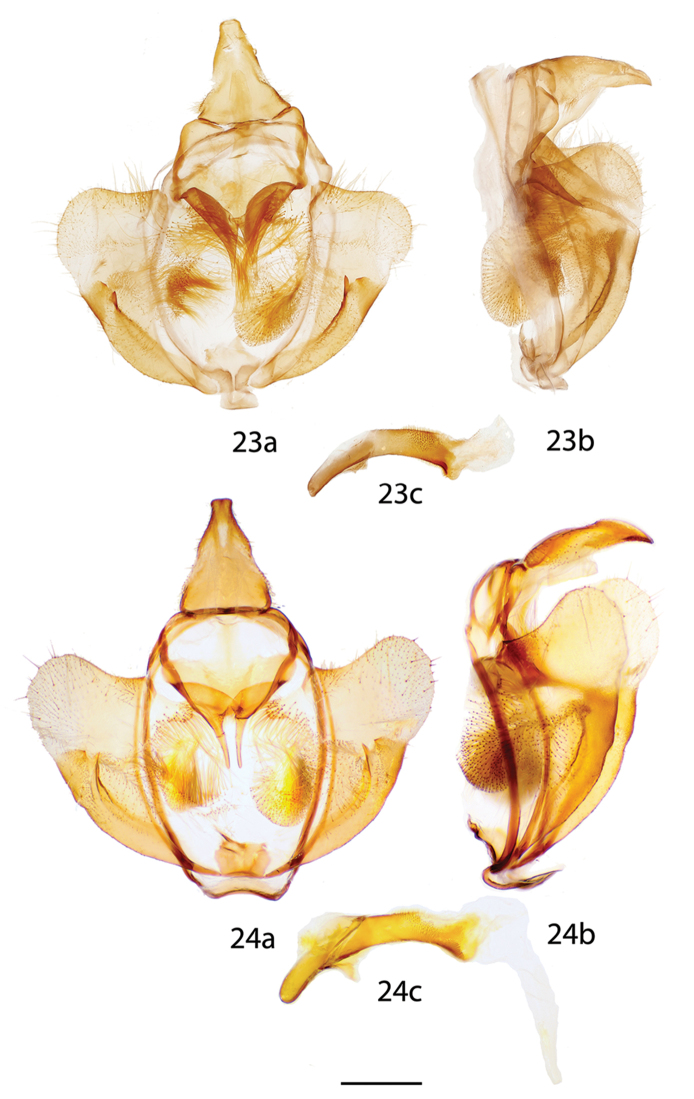
*Reinmara* male genitalia, **a** ventral **b** lateral **c** phallus lateral. **23** *R. enthona*, Suriname, Moengo, Boven Cottica River, St Laurent diss.: 10-25-15:2 (CUIC). **24** *R. atlantica* Holotype, Brazil, Espírito Santo, Linhares, 40 m, St Laurent diss.: 11-1-16:6 (DZUP). Scale bar: 1 mm.

**Figures 25–27.**
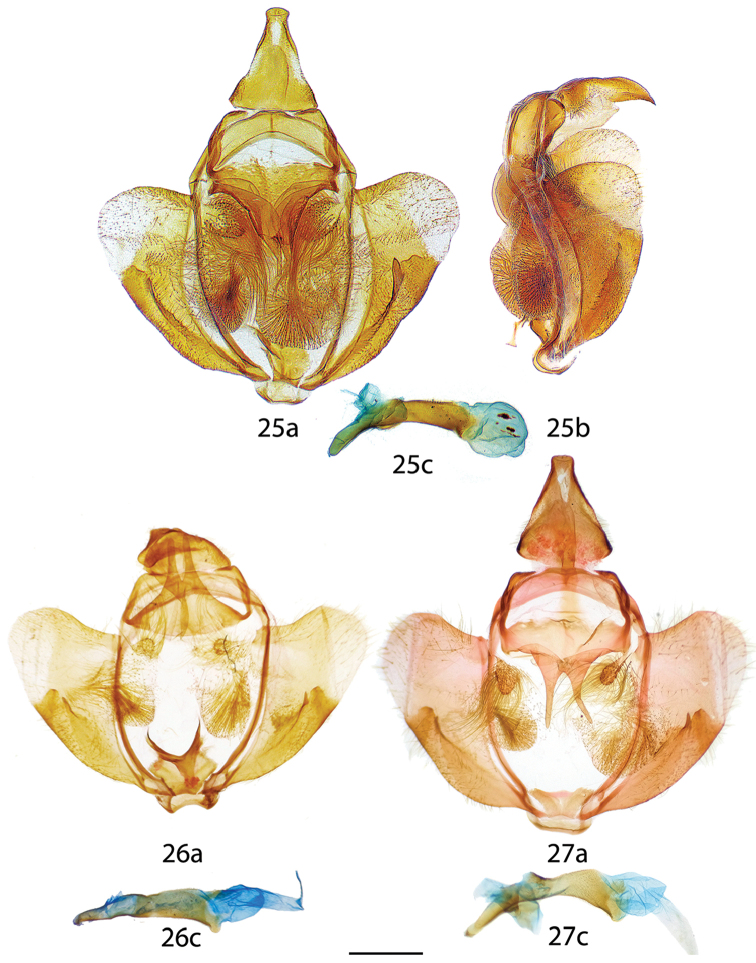
*Reinmara* male genitalia, **a** ventral **b** lateral **c** phallus lateral. **25** *R. andensis* Holotype, Bolivia, N. Yungas, 1000–1800 m, D. Herbin genitalia prep. H. 1134 (MNHN) **26** *R. occidentalis* Holotype, Ecuador, El Oro, 10 km NW of Piñas, 750 m, genitalia prep. 29.218 [phallus flipped horizontally, oriented somewhat dorsally] (MWM) **27** *R. occidentalis* Paratype, Ecuador, road Piñas to Saracay, 800 m, genitalia prep. 30.813 [phallus flipped horizontally] (MWM). Scale bar: 1 mm.

**Figures 28–30.**
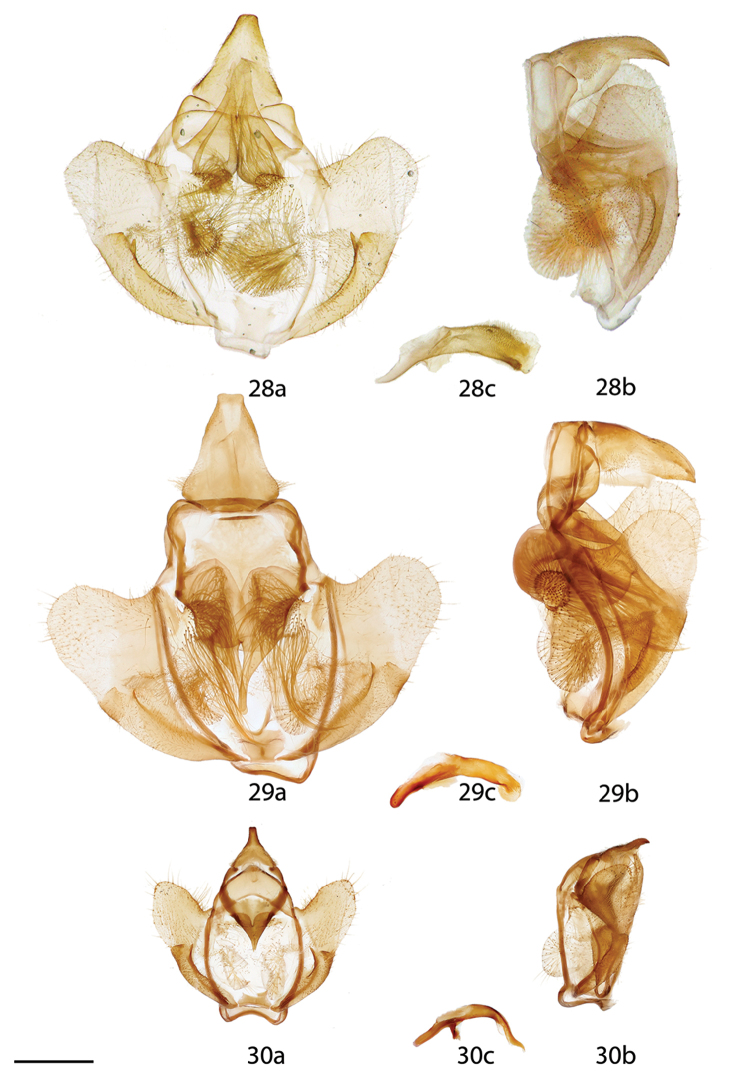
*Reinmara* male genitalia, **a** ventral **b** lateral **c** phallus lateral. **28** *R. wolfei*, Brazil, Distrito Federal, Planaltina, 1000 m, C. Mielke genitalia prep. 6.812 (CPAC) **29** *R. minasa*, Brazil, Espírito Santo, St Laurent diss.: 5-15-16:1 (CUIC) **30** *R. ignea* Paratype, Brazil, Santa Catarina, São Bento do Sul, Rio Vermelho, 968 m, St Laurent diss.: 5-6-16:1 (ISEZ). Scale bar: 1 mm.

**Figures 31–33.**
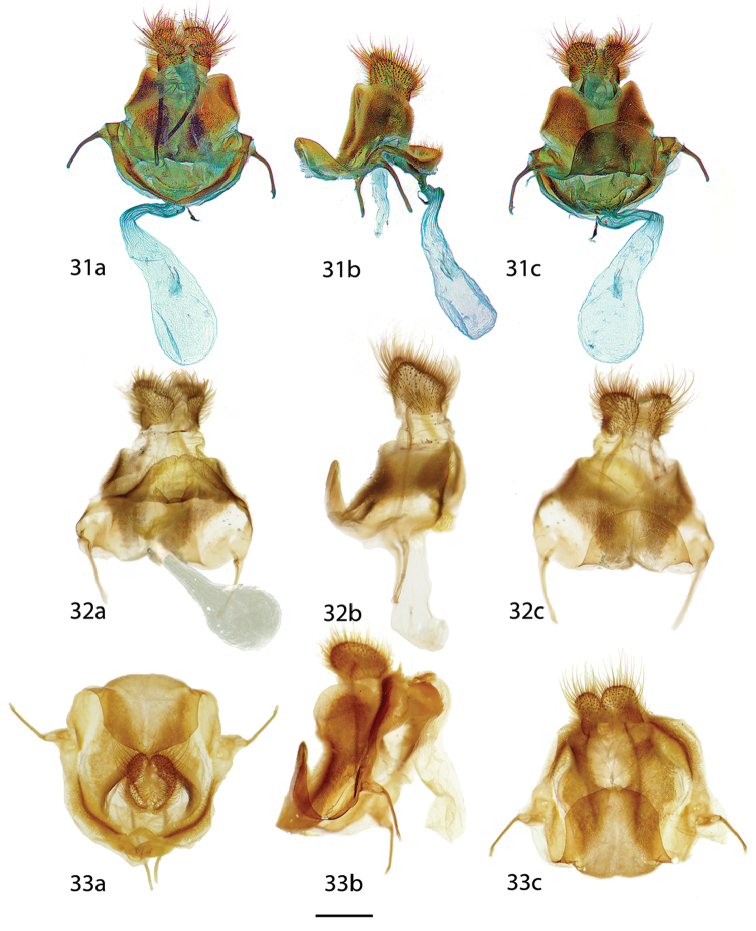
*Reinmara* female genitalia, **a** ventral **b** lateral **c** dorsal. **31** *R. enthona*, French Guiana, Kaw Rd., PK 37.5 + 2, 200 m, D. Herbin genitalia prep. H. 1103 (CDH) **32** *R. wolfei*, Brazil, Distrito Federal, Planaltina, 1000 m, C. Mielke genitalia prep. 6.879 (CPAC) **33** *R. minasa*, Brazil, São Paulo, Santo Antônio do Pinhal, Eugênio Lefèvre, 1200 m, C. Mielke genitalia prep. 28.071 (MZSP). Scale bar: 1 mm.

**Figures 34, 35.**
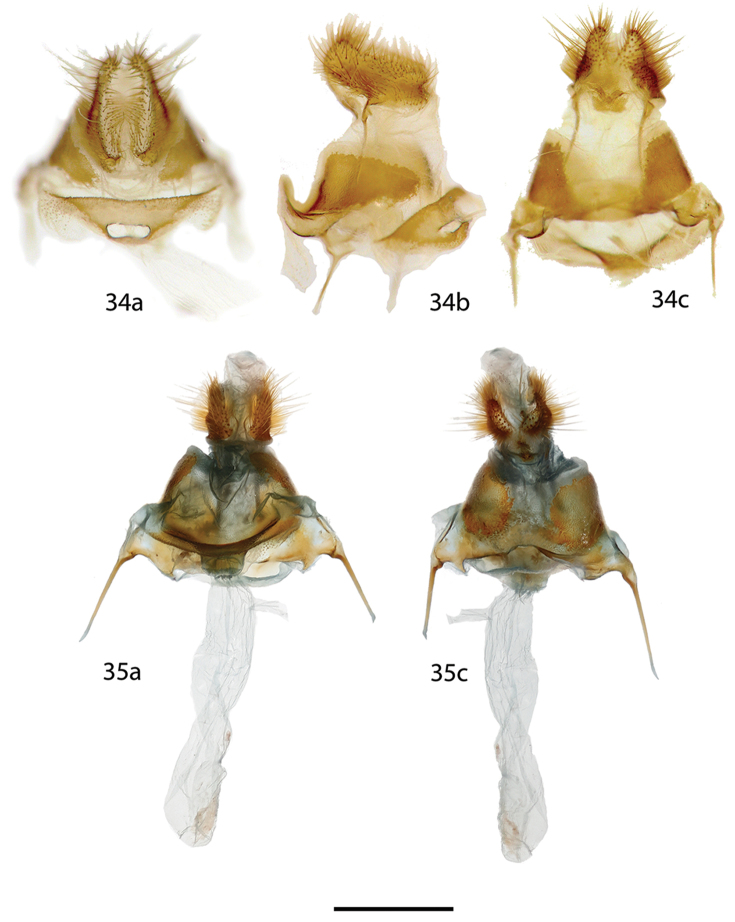
*Reinmara ignea* female genitalia, **a** ventral **b** lateral **c** dorsal. **34** Holotype ♀, Brazil, São Bento do Sul, Rio Natal, 550 m, C. Mielke genitalia prep. 20.982 (DZUP) **35** Paratype ♀, Brazil, Rio de Janeiro, Nova Friburgo, 1100 m, St Laurent diss.: 2-29-16:1 (USNM). Note: different orientation of two preparations obfuscates their actual similarity, in Fig. 34a the lamella ante- and postvaginalis are pressed downward. Scale bar: 1 mm.

**Figure 36.**
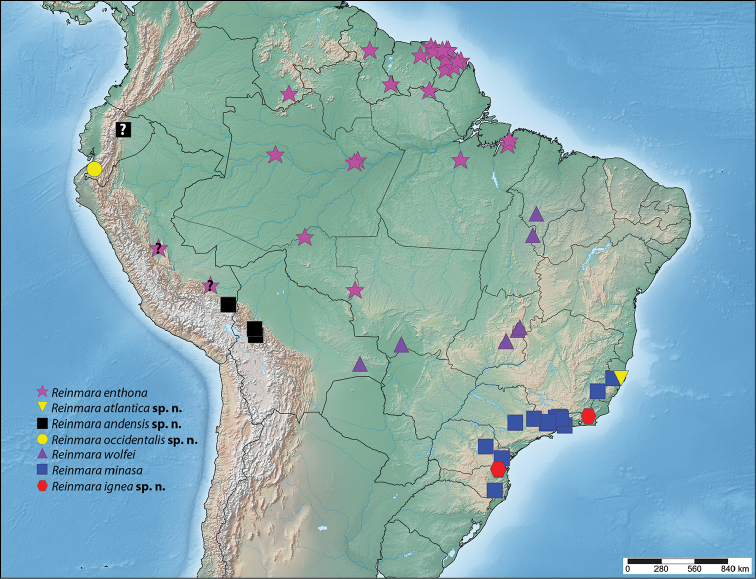
Known distribution of *Reinmara*.

## Supplementary Material

XML Treatment for Reinmara

XML Treatment for Reinmara enthona

XML Treatment for Reinmara atlantica

XML Treatment for Reinmara andensis

XML Treatment for Reinmara occidentalis

XML Treatment for Reinmara wolfei

XML Treatment for Reinmara minasa

XML Treatment for Reinmara ignea
